# THE USE OF ELDERCARING COORDINATION FOR RESOLVING CASES INVOLVING OLDER ADULTS AND HIGH-CONFLICT FAMILY DYNAMICS

**DOI:** 10.1093/geroni/igz038.2134

**Published:** 2019-11-08

**Authors:** Pamela B Teaster, Megan L Dolbin-MacNab

**Affiliations:** Virginia Tech, Blacksburg, Virginia, United States

## Abstract

The Association for Conflict Resolution and The Florida Chapter of the Association of Family and Conciliation Courts developed a model of Eldercaring Coordination for use in guardianship/probate cases involving high-conflict family dynamics that interfere with the well-being and safety of an older adult, limit adherence to court orders, impede court processes, or detract from the efficacy of guardianship and other appointments by the court. Developed by 40 organizations and entities in the United States and Canada, Eldercaring Coordination focuses on improving family dynamics so that the older adult, family members, and other involved parties can better work together and with professionals to make thoughtful and informed decisions and to support each other during times of transition. The purpose of this research was to gather information about participant experiences with Eldercaring Coordination. A pre-post test design was employed in which data were collected from older adults or their surrogates, family members and other court-ordered participants, judges and court administrators, and the Eldercaring Coordinators themselves. Findings from post-tests of 23 judges and court administrators revealed that the most common advantages of Eldercaring Coordination were that the intervention prioritized the older adults’ needs and improved family relationships. Post-test surveys from 17 Eldercaring Coordinators indicated some positive outcomes for older adults and their families, but also a need for enhanced authority, greater support from attorneys, and more cooperation from participants. Preliminary findings support the assertion that Eldercaring Coordination holds promise for intervening in high-conflict court cases involving older adults.

